# Oncogenic Mutations of p110α Isoform of PI 3-Kinase Upregulate Its Protein Kinase Activity

**DOI:** 10.1371/journal.pone.0071337

**Published:** 2013-08-01

**Authors:** Christina M. Buchanan, James M. J. Dickson, Woo-Jeong Lee, Mark A. Guthridge, Jackie D. Kendall, Peter R. Shepherd

**Affiliations:** 1 Department of Molecular Medicine, University of Auckland, Auckland, New Zealand; 2 Maurice Wilkins Centre for Molecular Biodiscovery, University of Auckland, Auckland, New Zealand; 3 School of Biological Sciences, University of Auckland, Auckland, New Zealand; 4 Australian Centre for Blood Diseases, Monash University, Melbourne, Victoria, Australia; 5 Auckland Cancer Society Research Centre, University of Auckland, Auckland, New Zealand; Dresden University of Technology, Germany

## Abstract

In addition to lipid kinase activity, the class-I PI 3-kinases also function as protein kinases targeting regulatory autophosphorylation sites and exogenous substrates. The latter include a recently identified regulatory phosphorylation of the GM-CSF/IL-3 βc receptor contributing to survival of acute myeloid leukaemia cells. Previous studies suggested differences in the protein kinase activity of the 4 isoforms of class-I PI 3-kinase so we compared the ability of all class-I PI 3-kinases and 2 common oncogenic mutants to autophosphorylate, and to phosphorylate an intracellular fragment of the GM-CSF/IL-3 βc receptor (βic). We find p110α, p110β and p110γ all phosphorylate βic but p110δ is much less effective. The two most common oncogenic mutants of p110α, H1047R and E545K have stronger protein kinase activity than wildtype p110α, both in terms of autophosphorylation and towards βic. Importantly, the lipid kinase activity of the oncogenic mutants is still inhibited by autophosphorylation to a similar extent as wildtype p110α. Previous evidence indicates the protein kinase activity of p110α is Mn^2+^ dependent, casting doubt over its role *in vivo*. However, we show that the oncogenic mutants of p110α plus p110β and p110γ all display significant activity in the presence of Mg^2+^. Furthermore we demonstrate that some small molecule inhibitors of p110α lipid kinase activity (PIK-75 and A66) are equally effective against the protein kinase activity, but other inhibitors (e.g. wortmannin and TGX221) show different patterns of inhibition against the lipid and protein kinases activities. These findings have implications for the function of PI 3-kinase, especially in tumours carrying p110α mutations.

## Introduction

The class 1 phosphoinositide 3-kinases (PI 3-kinases) play a critical role in pathways regulating functions such as cell metabolism, cell growth and survival, cytoskeletal rearrangements and cell movement [1], [2]. The class 1a PI 3-kinases are heterodimers consisting of a regulatory (or adapter) subunit (most commonly p85α, but also p85β, p55α, p50α or p55γ) coupled to a 110 kDa catalytic subunit (p110α, β, or δ). The class 1b PI3-K is also a dimer composed of a regulatory subunit (p101, p84 or p87PIKAP) coupled to the catalytic subunit (p110γ) [3]–[5]. Recently a range of oncogenic mutations have been identified in PIK3CA (p110α) and PIK3R1 (p85α) [6], [7] and these result in elevation of the lipid kinase activity [8], [9].

The PI 3-kinases are named for their lipid kinase activity, phosphorylating the 3′ position of the inositol ring in phosphatidylinositol (PtdIns) lipids and the consequences of this activity are well defined [10]–[12]. However PI 3-kinases are also known to possess protein kinase activity, with the ability to phosphorylate their own subunits [13], [14]. Evidence has been presented that the intrinsic phosphorylation of PI 3-kinase on Ser608 of the regulatory p85α subunit represents a form of negative feedback regulation [14]. This phosphorylation of Ser608 is stimulated by class 1a agonists [14], [15] and yet was observed to result in a dose-dependent decrease in PI 3-K lipid kinase activity [13], [14], an effect which was reversible by treatment with protein phosphatase 2A [13], [14] and alkaline phosphatase [13]. Studies with recombinant forms of PI 3-kinase indicate that there are differences in the protein kinase activity of different isoforms, for example 110α has a greater ability to phosphorylate p85 than p110β does [15]. Very little is known about the effect of oncogenic mutations in p110α on protein kinase activity, except that they retain the ability to phosphorylate Ser608 in p85α [8]. However, there has not been any detailed side-by-side comparison of the protein kinase activity of all PI 3-kinase isoforms, nor the susceptibility of this activity to different inhibitors. Interestingly some small molecules have been shown to differentially inhibit the lipid and protein kinase activities of PI 3-kinases [16], [17] raising the possibility that some of the new drugs being developed to target PI 3-kinases may do the same.

Exogenous targets of the protein kinase activity have also been identified. These include IRS-1 [18]–[20], MEK-1 [21], PDE3B [22] and 4EBP1 and H-Ras [15]. In most cases the functional consequences of these phosphorylation events have not been investigated but a recent study has provided evidence that phosphorylation of the GM-CSF/IL-3 βc receptor by p110α is functionally important in regulating cell survival in acute myeloid leukaemia cells [23]. This highlights the need to better understand the nature of the PI 3-kinase protein kinase activity towards exogenous substrates since it may play a role in normal cell regulation and/or tumourogenesis.

One argument against an *in vivo* role for the protein kinase activity of PI 3-kinase is that some studies to date have indicated it is manganese rather than magnesium dependent [13]–[15] and while magnesium is the most abundant divalent cation in cells [24], manganese is only present as a trace element [25].

Therefore to better understand the protein kinase activity of PI 3-kinase we have undertaken a comparison of the relative protein kinase activities of all the Class I PI 3-kinases as well as two common p110α oncogenic mutants (H1047R and E545K). These studies compared both the autophosphorylation and the exogenous kinase activity towards βic. Activities were determined in the presence of either Mn^2+^ or Mg^2+^ and we have also compared the effects on protein kinase activity of a range of known PI 3-kinase lipid kinase inhibitors.

Our studies provide the first evidence that oncogenic mutations of the p110α isoform of PI 3-kinase cause an upregulation of its protein kinase activity under physiologically relevant conditions. We describe distinct differences between wildtype and mutant p110α in relation to both the levels of p85α and p110 phosphorylation in buffers containing physiologically relevant Mg^2+^ concentrations, and the resulting impact on lipid kinase. We go on to show that the oncogenic forms of p110α also have increased protein kinase activity towards an exogenous substrate (βic). We further describe the protein kinase activity of the remaining Class I isoforms, elucidating the effects that this phosphorylation has on lipid kinase activity. These studies provide evidence that the protein kinase activity of class-I PI 3-kinase is capable of playing an important regulatory role in the cell and may contribute to the oncogenic potential of mutant forms of PI 3-kinase.

## Materials and Methods

### Recombinant PI 3-Kinase Synthesis

All Class 1a isoforms and mutants were produced in-house by co-expressing full-length human p85α with the indicated human full-length catalytic subunit in Sf9 cells infected with a recombinant baculovirus containing coding sequences for both the p85α (p85α; Genbank accession **NM_181523**) and Class 1a p110 subunits (p110α, Genbank accession **NM_006218**; p110β, **NM_006219**; p110δ, **NM_005026**) or Class 1b p110 subunit only (p110γ, **NM_002649**).

Site directed mutagenesis of p110α to yield the oncogenic mutants was performed by using either complementary (overlapping sense and antisense) oligonucleotides containing sequence mismatches incorporating the desired point mutation, or back to back phosphorylated primers spanning the region to be mutated (with one primer containing the desired point mutation). For both methods resultant plasmids were sequenced to confirm the insertion of the desired mutations prior to generation of recombinant baculovirus. All p110 constructs (wildtype and mutant) contain an N-His6 rTEV tag used to purify the complex by IMAC before final purification by anion exchange on MonoQ column. The N-His6-tag was removed by overnight cleavage with rTEV at 4°C, as this has been previously shown to impact protein kinase activity [26], [27].

### Recombinant βic Production

Production and purification of the histidine-tagged recombinant βic protein encompassing amino acids 445-881 of the intracellular domain of GM-CSF/IL-3 βc has been previously described [23], [28].

### Inhibitors

Wortmannin and LY294002 were from Sigma-Aldrich (St Louis, USA); TGX-221 was from Symansis (Auckland, NZ); PIK-75, A66 and AS252424 were synthesized in-house as previously described [29], [30].

### Protein Kinase Assays

Unless otherwise stated, protein kinase assays were carried out in a buffer containing 50 mM NaCl, 20 mM Tris/Cl (pH 7.4), 0.1 mM Na-orthovanadate, 12 µM ATP, 5 mM DTT, 2 µCi γ^ 33^P-ATP, and either 5 mM MgCl_2,_or 5 mM MnCl_2_ or both (as stated); Each reaction tube contained 0.5 µg kinase, 0.5 µg βic and inhibitors at stated concentrations. Unless otherwise stated, incubations were allowed to proceed for 20 minutes at 32°C and terminated by the addition of 5x electrophoresis sample buffer before complete denaturation at 99°C for 5 min. Components were separated by SDS PAGE, Coomassie-stained, dried and analysed by autoradiography (Molecular Dynamics Storm 680 PhosphorImager and quantified using ImageQuantTL software).

### PP2A Treatment

Where stated, kinases were dephosphorylated using the catalytic portion of PP2A (Sigma P1618 from bovine kidney); 1 μg of kinase was incubated with 0.3 U of PP2A for 15 minutes at 30°C in a buffer containing 4 mM CaCl_2_, 10 mM MgCl_2_, 5 mM MnCl_2_, 50 mM NaCl, 20 mM Tris/Cl (pH 7.4) and 5 mM DTT.

### Lipid Kinase Assays

To verify the impact of phosphorylation on lipid kinase activity, kinases were either pretreated with ATP (phosphorylated) or PP2A (unphosphorylated) before determining lipid kinase activity using phosphoinositol (PI) as a substrate. More specifically, kinases were either treated according to the Protein Kinase Assay (for 1 hour at 37°C without γ^33^P-ATP) or PP2A Treatment methods outlined above before the addition of EDTA to a final concentration of 2 mM EDTA; 10 μL of kinase (equivalent to 0.5 μg) was mixed with 90 μL buffer containing 40 mM Tris/Cl, 200 mM NaCl, 1 mM EDTA (pH 7.4). Each reaction point consisted of 20 μL of this kinase mixed with 10 μL of 1 mg/mL PI (Lipid Products, Surrey UK) in 10 mM Tris/Cl, 1 mM EDTA (pH 7.4), and 30 μL of ATP mix (10 mM MgCl_2_, 200 μM ATP, 1 μCi γ^33^P-ATP). The reaction was allowed to proceed for 1 hour at room temperature and stopped with 100 μL of 1 M HCl, before chloroform lipid extraction as previous (Method 2 described in [31]) with minor alterations described here. Specifically the re-extraction buffer consisted of 50:50 methanol/1 M HCl and the dried lipid was resuspended in 30 μL of chloroform: methanol (4:1 v/v). TLC plates were pre-treated with a solution containing 8 mM oxalic acid and 1 mM EDTA (pH 8) in MQ H_2_O/ethanol (3:1 v/v), and allowed to dry at room temperature overnight. Lipids were separated on the TLC plates using propan-1-ol/glacial acetic acid/MQ H_2_O (65:4:31 v/v). Assay results were analysed by autoradiography (Molecular Dynamics Storm 680 PhosphorImager and quantified using ImageQuantTL software).

### Lipid Kinase IC_50_ Determination

IC_50_ values were determined using the PI3K (human) HTRF Assay (Millipore, #33-016). All PI 3-K isoforms were made in-house and used in the range of their EC_65–80_ titration (18 ng/mL for H1047R, 6.5 ng/mL for E545K, 50 ng/mL for p110α, 400 ng/mL for p110β, 65 ng/ml for p110δ and 400 ng/mL for p110γ). Drugs were dissolved in DMSO and serially diluted in the same. Final DMSO concentration in assay was 2.5%.

## Results

### Characterisation of Lipid Kinase Activity in the Presence and Absence of PI 3-kinase Autophosphorylation

**Figure 1:**
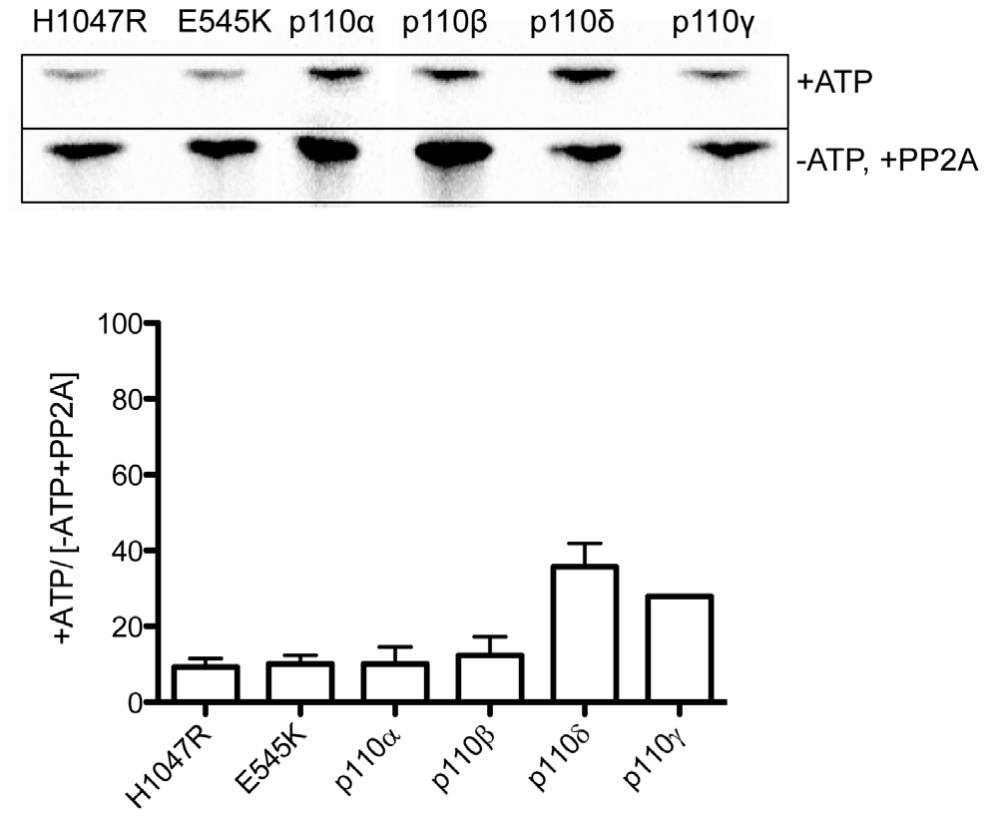
Effects of protein phosphorylation on lipid kinase conversion of PI to PI3P. Representative autoradiographs and graphs showing the level of ^33^P incorporated into PI3P in the presence and absence of ATP and PP2A (N≥2).

Previous studies using endogenous purified proteins have shown that phosphorylation of class-I PI 3-K reduces the lipid kinase activity of all isoforms except p110γ [13]–[15], [32], [33], although more recently a study comparing wildtype and mutant p110α reported that p85α phosphorylation had no impact on lipid kinase activity [34]. Here we have performed a comprehensive side-by-side comparison of all Class-I PI 3-kinases as well as two common oncogenic mutants of p110α (**Figure** **1**). Importantly the presence of epitope tags is known to affect the activity of recombinant PI 3-kinases [26], [27] so our recombinant proteins have the tag cleaved to minimize any such effects. Since it has been reported that there are pre-existing high levels of phosphorylation in the Sf9-produced recombinant protein [35], we first pre-treated the kinase with protein phosphatase 2A, and then carried out incubations in the presence and absence of ATP. These experiments showed a clear effect of protein phosphorylation on lipid kinase activity (**Figure 1**), with wildtype and mutant p110α and p110β exhibiting >80% knockdown in lipid kinase activity with phosphorylation, while p110δ and γ were less impacted. While these results contrast to the recent report of Layton *et al* (2012) [34], the results for p110α and p110β are in agreement with previous findings [13], [14], [33]. The moderate 60% knockdown of p110δ lipid kinase activity varies somewhat from previous findings [32] which demonstrated >90% knockdown of lipid kinase activity with autophosphorylation. Moreover previous studies suggested there would be no change in lipid kinase activity for p110γ following autophosphorylation [36], [37], and while p110γ was less impacted than p110α and β, in our hands it was still reduced to less than 40% activity. Importantly we find that the lipid kinase activity of p110α mutants is reduced following autophosphorylation by the same degree as wildtype p110α (**Figure 1**).

### Divalent Cation Dependence

**Figure 2:**
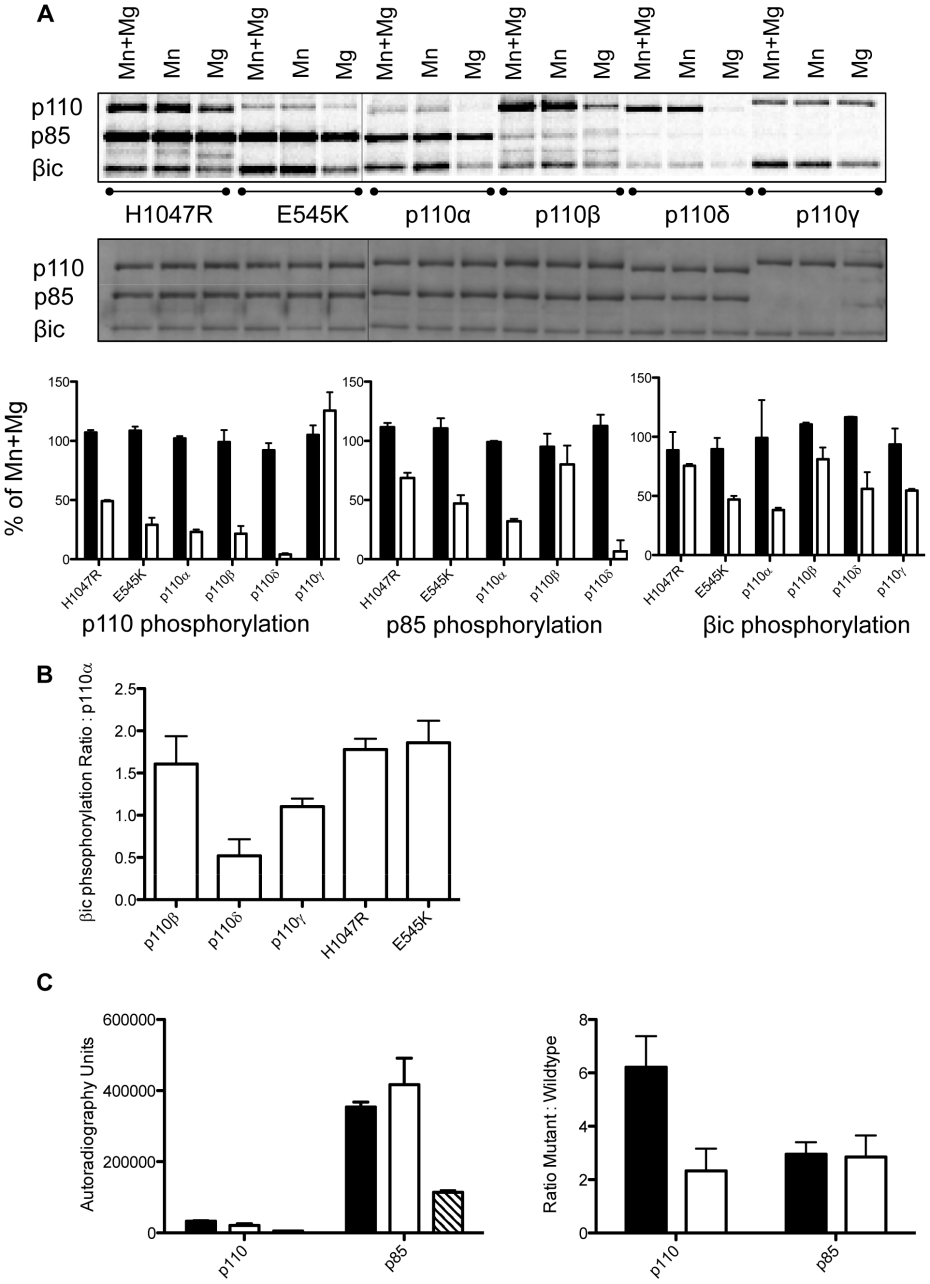
Effects of divalent cation on protein kinase activity. A) Representative autoradiograph scan showing relative intensity of phosphorylation in the presence of Mn^2+^ and Mg^2+^, Mn^2+^ alone, and Mg^2+^ alone. Graphs summarise the results of N = 5 individual experiments for Manganese (black bars) and Magnesium (white bars). B) Graph showing the abilities of different isoforms to phosphorylate βic, relative to p110α, N≥3 experiments. C) Graphs comparing the degree of autophosphorylation for the two oncogenic mutants, H1047R (black bars) and E545K (white bars) to p110α (hatched bars), N≥3 experiments.

One of the earliest reports of the protein kinase activity of PI 3-kinase showed that this activity was dependent on Mn^2+^ [14], and this was reiterated by Dhand *et al.* (1994) [13]. While subsequent studies have been carried out comparing the Mn/Mg dependent autophosphorylation of p110α and p110δ [32] and p110α and p110β [33], a comprehensive investigation of all the isoforms has never been completed. Furthermore, no studies have been carried out to test the ability of different PI 3-kinase isoforms to phosphorylate the exogenous substrate βic. Therefore we compared the protein kinase activities of all the isoforms and the two oncogenic mutants in the presence of Mn^2+^, Mg^2+^ or both (**Figure 2A**).

We observe autophosphorylation of p110 subunit by p110α wildtype and mutants, and p110β in the presence of Mg^2+^, albeit much reduced in comparison to kinase incubated with Mn^2+^, while p110γ retained 100% activity in Mg^2+^ only buffer. We only observe significant p85 phosphorylation in the proteins containing wildtype or oncogenic p110α (**Figure 2A**). Of particular interest, phosphorylation of the exogenous substrate βic was observed in the presence of Mg^2+^ for all isoforms but with the relative level of phosphorylation being different for the different isoforms. p110β and the oncogenic mutants exhibited the most activity (approaching 2-fold more activity than p110α), while p110 γ and p110α had equal activity, and p110δ very little (**Figure 2A and 2B**). The largest observable difference between the two oncogenic forms of PI 3-kinase was that autophosphorylation of p110 remained strong in the presence of Mg^2+^ for H1047R mutant but was not present in the E545K mutant and wildtype p110α (**Figure 2C**). The significance of this remains to be elucidated.

### Lipid Kinase Inhibitors also Inhibit the Protein Kinase Activity of PI 3-kinase

**Figure 3:**
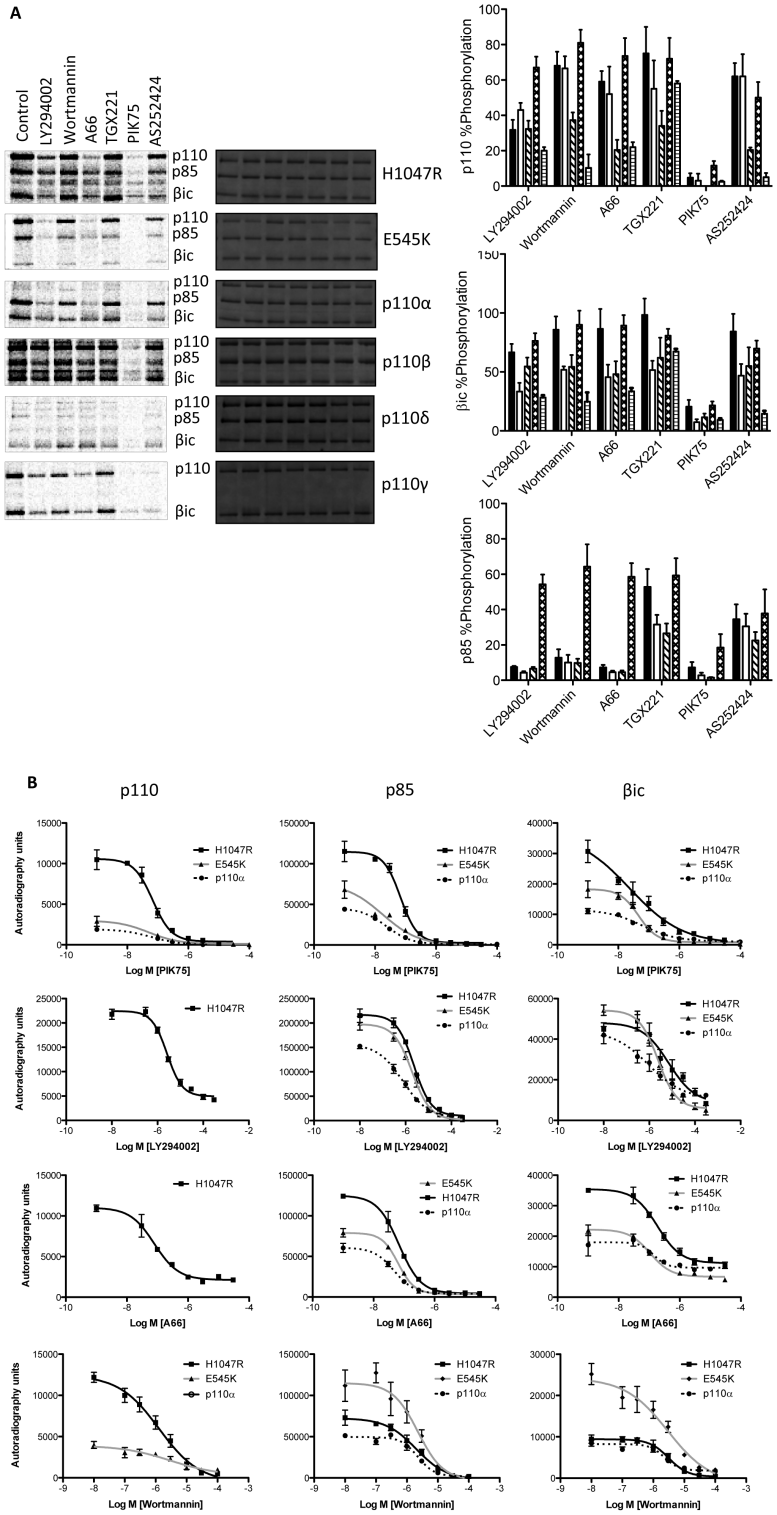
Effects of pan- and isoform-specific inhibitors on protein kinase activity. **A)** Representative autoradiograph scans showing relative intensity of phosphorylation in the presence of pan-inhibitors LY294002 and wortmannin, and p110α-specific inhibitors A66 and PIK-75, p110β-specific inhibitor TGX221 and p110γ-specific inhibitor AS252424. Graphs summarise the results of N≥3 experiments for H1047R (black bars) and E545K (white bars) to p110α (hatched bars), p110β (cross-hatched bars) and p110γ (horizontal stripe bars) B) IC_50_ curves for selected inhibitors PIK-75, LY294002, A66 and wortmannin against p110α, H1047R and E545K.

We also tested the effect of different small molecules known to inhibit the lipid kinase activity of PI 3-kinases. We first challenged all the isoforms and mutants with a fixed concentration of the pan-specific inhibitors LY294002 and wortmannin, p110α-specific inhibitors A66 and PIK-75, the p110β-specific inhibitor TGX221, and the p110γ inhibitor AS252424. All inhibitors were initially screened at a final concentration of 10 μM except LY294002, which was used at a final concentration of 100 μM. A66 and AS252424 follow the same pattern of isoform selectivity as seen in their lipid kinase activities (**Figure 3A**). Interestingly p110β protein kinase activity was largely resistant to all inhibitors except PIK-75, with TGX221 being notably ineffective despite it being known as a p110β selective lipid kinase inhibitor. Wortmannin was also surprisingly ineffective. Our results clearly showed that PIK-75 was the most effective inhibitor, knocking down the p110 and p85 phosphorylation of all kinases by ≥90% even though it was previously described as a p110α selective inhibitor (**Figure 3A**).

To further understand these results we generated IC_50_ inhibition curves for PIK-75, LY294002, A66 and wortmannin against p110α and the oncogenic mutants (**Figure 3B** and **Table 1**). Interestingly the IC_50_’s generated for the protein kinase and lipid kinase inhibition were broadly in the same range for PIK-75, LY294002 and A66 (**Table 1** and **2**). However, the IC_50_ of wortmannin against the protein kinase activity was significantly higher than the lipid kinase activity in the conditions tested (**Figure 3B**, **Table 1** and **2**).

**Table 1:** The IC50 of different small molecule inhibitors against the protein kinase activities of wildtype and mutant p110α.

	Protein Kinase IC50 (nM)								
	p110			p85			βic		
	H1047R	E545K	p110α	H1047R	E545K	p110α	H1047R	E545K	p110α
PIK-75	99	101	56	67	18	27	15	84	52
LY294002	2584	ND	ND	2267	1903	757	6111	2413	1151
A66	47	ND	ND	69	64	47	185	106	135
Wortmannin	4150	2320	ND	1960	2490	2220	3240	3480	2260

ND = no signal detected.

**Table 2:** The IC50 of different small molecule inhibitors against the lipid kinase activity of wildtype and mutant p110α.

	Lipid Kinase IC50 (nM)		
	H1047R	E545K	p110α
PIK-75	6	24	7
LY294002	1836	528	1986
A66	41	30	34
Wortmannin	3	3	3

## Discussion

PI 3-K has been recognized as a dual lipid/protein kinase since the seminal papers of Carpenter *et al*. (1993) and Dhand *et al.* (1994) [13], [14]. While the lipid kinase activity is acknowledged as critical for many cellular functions, the physiological significance of the protein kinase activity has long been questioned due to its Mn^2+^ dependency and lack of *in vivo* relevance. However, we have recently shown that PI 3-K protein kinase activity is deregulated in >80% of primary AML patient samples [23], [38] and that PI 3-K-mediated phosphorylation of the GM-CSF/IL-3 receptors regulates cell survival [23]. These findings have reinitiated interest in this area. Here we have systematically studied the *in vitro* protein kinase activity of all human class-1 PI 3-kinase isoforms as well as 2 common gain-of-function oncogenic mutants of p110α, the catalytic-domain H1047R mutant and the helical-domain mutant E545K. We further evaluate and discuss their ability to autophosphorylate as well as phosphorylate an exogenous substrate (GM-CSF/IL-3 βc receptor-derived βic peptide), in the presence and absence of Mn^2+^ and Mg^2+^, and the impact of a range of ATP-competitive inhibitors on their protein kinase activity.

With the exception of p110δ, our results show that all the forms of PI 3-K investigated retain some level of protein kinase activity in the presence of Mg^2+^ alone, with p110γ and the H1047R mutant retaining significantly more protein kinase activity than the other forms. Divalent cations such as Mg^2+^ and Mn^2+^ are required to coordinate the phosphates in ATP to give it the correct conformation for catalytic reactions [39], and while magnesium is the most abundant divalent cation in cells [24], manganese is only present as a trace element at μM concentrations, with free cytoplasmic concentrations further reduced due to intracellular compartmentalization [25]. Previous evidence indicated that the protein kinase activity of PI 3-K was Mn^2+^ dependent [13]–[15], which cast doubt over the *in vivo* role of this enzyme activity. Therefore our observation of protein kinase activity in the presence of only Mg^2+^ (especially the oncogenic mutants of p110α) provides evidence that this activity could be important *in vivo.*

All the forms of PI 3-K, again excepting p110δ, were effective to different degrees with regards to phosphorylating the exogenous substrate, βic. The oncogenic forms of PI 3-K have increased protein kinase activity compared to wildtype p110α, and these mutants, together with p110β and p110γ showed strong phosphorylation of βic. The increased protein kinase activity against an exogenous substrate indicates that these forms could possibly phosphorylate other substrates, including those contributing to oncogenesis. This could in part explain the oncogenic effects seen with overexpression of p110β and p110γ in cell models [40], [41], and why cellular overexpression of these alone can contribute to tumourogenesis [42], [43]. It is important to note that while we have shown that PI 3-K-mediated phosphorylation of the GM-CSF/IL-3 receptors regulates cell survival [23], it seems likely that other p110-mediated phosphorylation events also occur, since evidence has been presented that PI 3-kinase phosphorylates a range of signaling molecules including IRS-1 [18]–[20], 4EBP1 and H-Ras [15]. To date these phosphorylation events have been poorly characterized but it remains possible that these might contribute to activation of signaling pathways in cancer.

In general p110α and the E545K mutant showed strong p85 phosphorylation with little p110 autophosphorylation, while p110β and p110γ showed high p110 phosphorylation. The H1047R mutant maintained the strong p85 phosphorylation as seen with the wildtype PI 3-Kα isoform, but also showed elevated levels of p110 phosphorylation, approaching those of p110β. The significance of this increase in p110 phosphorylation in the H1047R mutant is not clear, especially as the lipid kinase activity of both oncogenic isoforms and wildtype p110α are inhibited by pre-phosphorylation to a similar extent. However since the level of reduction in the lipid kinase activity is similar in all three forms of p110α and p110β, it demonstrates, in agreement with Layton *et al* (2012) [34], that the increased lipid kinase activity in H1047R and E545K are not due to any reduction in auto-regulatory protein kinase activity. This further reinforces the hypothesis that the area where the increased protein kinase activity might have the most impact on cellular function is in the phosphorylation of exogenous substrates.

Our observations regarding the potency of PIK-75 relative to other inhibitors (TGX221 and AS252424) support our previous findings showing PIK-75 preferential inhibition of Ser585 phosphorylation in the GM-CSF/IL-3 receptors [23]. The fact that the inhibition of lipid and protein kinase activity by small molecules does not always directly correlate is also interesting and has potential implications for the use of PI 3-K inhibitors in the clinic, as it may explain the functional differences between different inhibitors in some contexts. For example, our finding that LY294002 is more effective at blocking the protein kinase activity of PI 3-K relative to wortmannin supports the unexpected observation that LY294002 was more effective than wortmannin at blocking the anti-apoptotic effect of GM-CSF [44]. However detailed explanation for the observed differences will require in depth structural studies to define how protein substrates interact with the kinase domain.

In summary, we compared the *in vitro* protein kinase activity of all human class-1 PI 3-kinase isoforms as well as 2 common gain-of-function oncogenic mutants of p110α, H1047R and E545K. We found that the oncogenic mutants have stronger protein kinase activity as measured by autophosphorylation, as well as phosphorylation of the exogenous substrate, βic. Furthermore we show that the oncogenic mutants (especially H1047R) are not as reliant on Mn^2+^ and retain significantly more protein kinase activity in the presence of Mg^2+^ alone. We demonstrate that the protein kinase activity of the wildtype and oncogenic forms of p110α are all inhibited by small molecules known to inhibit the lipid kinase activity of p110α; including LY294002, A66 and to a lesser degree wortmannin. However, inhibition was particularly pronounced with PIK-75. In addition we showed that p110β and p110γ exogenous and auto-phosphorylation was retained in the presence of Mg^2+^, and these isoforms were relatively resistant to inhibition by all inhibitors except PIK-75 (and AS252424 for p110γ). In contrast p110δ was Mn^2+^ dependent, only weakly phosphorylated βic and was fully suppressed by all inhibitors used. Since PI 3-K phosphorylates exogenous substrates and has been linked to changes in lipid kinase activity and possible activation of alternate signaling pathways [45], this increased protein kinase activity of E545K and especially H1047R in the presence of Mg^2+^ could have implications for the physiological activity of PI 3-K, especially in tumours carrying these mutations.
